# Supplementary material for: Hot spot prediction in protein-protein interactions by an ensemble system
Source: BMC Syst Biol. 2018 Dec 31;12(Suppl 9):132. doi: 10.1186/s12918-018-0665-8 (PMC6311905; doi:10.1186/s12918-018-0665-8)
Supplement: Supplementary file 2 — All relevant MATLAB code. A simple Matlab implement of our predictor. (ZIP 19,742 kb) [file 12918_2018_665_MOESM2_ESM.zip › Additional file 2/Read ASA/ASA website/dbMPIKT/4FZAB.html]

 NetSurfP - Protein Surface Accessibility and Secondary Structure Predictions

  

|  |  |  |
| --- | --- | --- |
|  |  | NetSurfP - Protein Surface Accessibility and Secondary Structure Predictions  Technical University of Denmark |

  


---

```
# For publication of results, please cite:
# A generic method for assignment of reliability scores applied to solvent accessibility predictions.
# Bent Petersen, Thomas Nordahl Petersen, Pernille Andersen, Morten Nielsen and Claus Lundegaard
# BMC Structural Biology 2009, 9:51 doi:10.1186/1472-6807-9-51
#
# Column 1: Class assignment - B for buried or E for Exposed - Threshold: 25% exposure, but not based on RSA
# Column 2: Amino acid
# Column 3: Sequence name
# Column 4: Amino acid number
# Column 5: Relative Surface Accessibility - RSA
# Column 6: Absolute Surface Accessibility
# Column 7: Z-fit score for RSA prediction
# Column 8: Probability for Alpha-Helix
# Column 9: Probability for Beta-strand
# Column 10: Probability for Coil
E G  4FZAB                  1    0.901  70.893  -1.382   0.003   0.003   0.994
E A  4FZAB                  2    0.704  77.548  -1.367   0.018   0.019   0.964
E M  4FZAB                  3    0.554 110.855  -1.687   0.018   0.019   0.964
E A  4FZAB                  4    0.609  67.112  -0.073   0.058   0.017   0.925
E D  4FZAB                  5    0.466  67.151   0.116   0.053   0.005   0.942
B P  4FZAB                  6    0.162  23.059  -0.036   0.600   0.003   0.397
E E  4FZAB                  7    0.563  98.321   0.630   0.694   0.003   0.303
E E  4FZAB                  8    0.601 105.012   0.988   0.717   0.014   0.269
E L  4FZAB                  9    0.322  58.903   1.226   0.751   0.050   0.199
B F  4FZAB                 10    0.073  14.731   0.752   0.701   0.107   0.192
E T  4FZAB                 11    0.571  79.239   1.428   0.662   0.225   0.113
E K  4FZAB                 12    0.373  76.829   1.178   0.594   0.285   0.121
B L  4FZAB                 13    0.191  34.899   0.771   0.453   0.248   0.299
E E  4FZAB                 14    0.445  77.724   0.740   0.321   0.252   0.427
E R  4FZAB                 15    0.269  61.693   1.190   0.252   0.423   0.325
B I  4FZAB                 16    0.064  11.840   0.325   0.148   0.418   0.435
B G  4FZAB                 17    0.171  13.473  -1.008   0.066   0.296   0.638
E K  4FZAB                 18    0.528 108.527   0.450   0.019   0.141   0.840
B G  4FZAB                 19    0.248  19.541  -2.411   0.018   0.088   0.893
B S  4FZAB                 20    0.202  23.639  -1.355   0.056   0.142   0.802
E F  4FZAB                 21    0.311  62.438  -0.983   0.066   0.296   0.638
B G  4FZAB                 22    0.069   5.407  -0.874   0.022   0.552   0.426
B E  4FZAB                 23    0.224  39.185   1.397   0.018   0.846   0.136
B V  4FZAB                 24    0.026   3.981   0.746   0.006   0.962   0.032
B F  4FZAB                 25    0.121  24.204   1.310   0.001   0.959   0.040
B K  4FZAB                 26    0.133  27.317   1.476   0.000   0.983   0.017
B G  4FZAB                 27    0.018   1.377   0.488   0.000   0.983   0.017
B I  4FZAB                 28    0.192  35.612   1.497   0.000   0.983   0.017
B D  4FZAB                 29    0.162  23.344   0.417   0.001   0.959   0.040
B N  4FZAB                 30    0.256  37.464   0.851   0.004   0.514   0.481
E R  4FZAB                 31    0.681 155.857  -0.079   0.004   0.197   0.799
E T  4FZAB                 32    0.587  81.375   0.293   0.005   0.045   0.951
E Q  4FZAB                 33    0.546  97.498   0.060   0.005   0.045   0.951
E Q  4FZAB                 34    0.351  62.635   1.564   0.004   0.514   0.481
B V  4FZAB                 35    0.214  32.907   1.956   0.001   0.959   0.040
B V  4FZAB                 36    0.056   8.607   1.857   0.001   0.959   0.040
B A  4FZAB                 37    0.020   2.171   0.917   0.001   0.959   0.040
B I  4FZAB                 38    0.048   8.824   0.766   0.000   0.983   0.017
B K  4FZAB                 39    0.082  16.785   0.534   0.000   0.983   0.017
B I  4FZAB                 40    0.186  34.392   1.109   0.000   0.983   0.017
B I  4FZAB                 41    0.063  11.636   0.960   0.001   0.959   0.040
E D  4FZAB                 42    0.475  68.419   1.538   0.002   0.816   0.182
B L  4FZAB                 43    0.111  20.306   0.138   0.005   0.262   0.733
E E  4FZAB                 44    0.740 129.261   0.956   0.005   0.045   0.951
E E  4FZAB                 45    0.657 114.708   0.626   0.018   0.019   0.964
E A  4FZAB                 46    0.413  45.546   0.316   0.016   0.005   0.979
E E  4FZAB                 47    0.679 118.639   0.713   0.858   0.002   0.139
E D  4FZAB                 48    0.690  99.386   1.304   0.923   0.002   0.076
E E  4FZAB                 49    0.314  54.786   1.027   0.923   0.002   0.076
B I  4FZAB                 50    0.180  33.245   0.672   0.970   0.001   0.030
E E  4FZAB                 51    0.604 105.501   1.340   0.970   0.001   0.030
E D  4FZAB                 52    0.425  61.214   2.063   0.988   0.000   0.012
B I  4FZAB                 53    0.021   3.885   1.046   0.988   0.000   0.012
E Q  4FZAB                 54    0.309  55.187   1.360   0.988   0.000   0.012
E Q  4FZAB                 55    0.433  77.423   1.626   0.970   0.001   0.030
B E  4FZAB                 56    0.057   9.888  -0.013   0.988   0.000   0.012
B I  4FZAB                 57    0.049   9.139   0.632   0.988   0.000   0.012
E T  4FZAB                 58    0.481  66.784   1.752   0.988   0.000   0.012
B V  4FZAB                 59    0.100  15.355   0.769   0.970   0.001   0.030
B L  4FZAB                 60    0.085  15.509   0.198   0.970   0.001   0.030
E S  4FZAB                 61    0.432  50.595   0.578   0.858   0.002   0.139
E Q  4FZAB                 62    0.553  98.712   1.017   0.600   0.003   0.397
B C  4FZAB                 63    0.078  10.895   0.617   0.176   0.004   0.820
E D  4FZAB                 64    0.668  96.331   0.268   0.016   0.005   0.979
B S  4FZAB                 65    0.168  19.643   0.076   0.003   0.003   0.994
E S  4FZAB                 66    0.604  70.765   0.994   0.018   0.019   0.964
B Y  4FZAB                 67    0.226  48.211  -0.164   0.052   0.084   0.864
B V  4FZAB                 68    0.039   5.994   0.694   0.069   0.386   0.545
B T  4FZAB                 69    0.059   8.156   0.600   0.021   0.756   0.223
E K  4FZAB                 70    0.314  64.652   1.018   0.018   0.846   0.136
B Y  4FZAB                 71    0.054  11.604   0.534   0.018   0.846   0.136
B Y  4FZAB                 72    0.178  38.060   1.158   0.021   0.756   0.223
B G  4FZAB                 73    0.139  10.971  -1.467   0.018   0.846   0.136
B S  4FZAB                 74    0.076   8.942  -0.014   0.011   0.918   0.071
B Y  4FZAB                 75    0.164  34.983  -0.003   0.001   0.959   0.040
E L  4FZAB                 76    0.355  65.037   0.563   0.001   0.959   0.040
E K  4FZAB                 77    0.440  90.549   0.719   0.003   0.718   0.279
E G  4FZAB                 78    0.717  56.452   0.477   0.005   0.045   0.951
E S  4FZAB                 79    0.568  66.534   0.209   0.005   0.015   0.979
B K  4FZAB                 80    0.231  47.476   1.924   0.002   0.816   0.182
B L  4FZAB                 81    0.025   4.486   1.099   0.001   0.959   0.040
B W  4FZAB                 82    0.064  15.296   1.277   0.006   0.962   0.032
B I  4FZAB                 83    0.019   3.515   1.640   0.001   0.959   0.040
B I  4FZAB                 84    0.019   3.570   1.380   0.001   0.959   0.040
B M  4FZAB                 85    0.023   4.682   1.332   0.001   0.900   0.099
B E  4FZAB                 86    0.053   9.312   0.143   0.001   0.900   0.099
B Y  4FZAB                 87    0.083  17.780   0.242   0.004   0.616   0.381
B L  4FZAB                 88    0.040   7.361   0.166   0.018   0.088   0.893
E G  4FZAB                 89    0.492  38.681  -0.163   0.005   0.015   0.979
E G  4FZAB                 90    0.346  27.191  -1.670   0.058   0.017   0.925
B G  4FZAB                 91    0.204  16.031  -1.245   0.115   0.016   0.868
B S  4FZAB                 92    0.194  22.725  -0.285   0.430   0.016   0.555
B A  4FZAB                 93    0.035   3.846  -0.494   0.923   0.002   0.076
E L  4FZAB                 94    0.284  52.055   0.952   0.970   0.001   0.030
E D  4FZAB                 95    0.402  57.971   1.096   0.970   0.001   0.030
B L  4FZAB                 96    0.104  18.987   1.126   0.923   0.002   0.076
B L  4FZAB                 97    0.077  14.172   0.213   0.923   0.002   0.076
E R  4FZAB                 98    0.571 130.805   1.349   0.782   0.003   0.216
E A  4FZAB                 99    0.649  71.531   0.715   0.246   0.004   0.750
E G  4FZAB                100    0.429  33.770   0.449   0.053   0.005   0.942
E P  4FZAB                101    0.571  80.968   0.506   0.005   0.015   0.979
B F  4FZAB                102    0.055  11.139  -0.093   0.005   0.015   0.979
E D  4FZAB                103    0.559  80.581   1.303   0.003   0.003   0.994
B E  4FZAB                104    0.120  20.999  -0.373   0.923   0.002   0.076
E F  4FZAB                105    0.588 118.112   0.822   0.970   0.001   0.030
B Q  4FZAB                106    0.172  30.773   1.543   0.970   0.001   0.030
B I  4FZAB                107    0.015   2.756   1.329   0.970   0.001   0.030
B A  4FZAB                108    0.074   8.133   0.978   0.970   0.001   0.030
E T  4FZAB                109    0.309  42.886   1.731   0.988   0.000   0.012
B M  4FZAB                110    0.048   9.705   1.480   0.970   0.001   0.030
B L  4FZAB                111    0.018   3.204   1.451   0.988   0.000   0.012
B K  4FZAB                112    0.231  47.578   1.653   0.988   0.000   0.012
B E  4FZAB                113    0.135  23.637   0.435   0.988   0.000   0.012
B I  4FZAB                114    0.025   4.680   1.242   0.988   0.000   0.012
B L  4FZAB                115    0.033   6.097  -0.037   0.988   0.000   0.012
E K  4FZAB                116    0.292  60.003   1.271   0.970   0.001   0.030
B G  4FZAB                117    0.033   2.589  -0.633   0.970   0.001   0.030
B L  4FZAB                118    0.036   6.500   0.190   0.970   0.001   0.030
E D  4FZAB                119    0.328  47.322   1.035   0.970   0.001   0.030
B Y  4FZAB                120    0.202  43.125   0.712   0.970   0.001   0.030
B L  4FZAB                121    0.042   7.617   0.500   0.938   0.007   0.055
B H  4FZAB                122    0.205  37.344   0.846   0.782   0.003   0.216
E S  4FZAB                123    0.733  85.966   0.952   0.406   0.004   0.590
E E  4FZAB                124    0.538  94.076   1.233   0.053   0.005   0.942
E K  4FZAB                125    0.529 108.856   0.027   0.005   0.015   0.979
B K  4FZAB                126    0.164  33.735   1.115   0.004   0.197   0.799
B I  4FZAB                127    0.105  19.388   0.548   0.022   0.552   0.426
B H  4FZAB                128    0.151  27.467   0.663   0.021   0.451   0.528
B R  4FZAB                129    0.231  52.784   0.276   0.066   0.296   0.638
B D  4FZAB                130    0.177  25.578  -0.689   0.056   0.142   0.802
B I  4FZAB                131    0.068  12.525   0.817   0.052   0.084   0.864
E K  4FZAB                132    0.307  63.232   0.979   0.053   0.043   0.903
B A  4FZAB                133    0.101  11.141  -0.753   0.053   0.043   0.903
E A  4FZAB                134    0.335  36.928  -0.129   0.018   0.047   0.935
B N  4FZAB                135    0.102  14.991  -0.888   0.064   0.216   0.721
B V  4FZAB                136    0.057   8.761   0.684   0.023   0.655   0.322
B L  4FZAB                137    0.072  13.110  -0.325   0.021   0.756   0.223
B L  4FZAB                138    0.084  15.307   0.930   0.023   0.655   0.322
B S  4FZAB                139    0.122  14.345   0.139   0.020   0.205   0.775
E E  4FZAB                140    0.610 106.619   0.085   0.018   0.047   0.935
E Q  4FZAB                141    0.710 126.752   0.675   0.018   0.019   0.964
B G  4FZAB                142    0.155  12.175  -0.253   0.018   0.019   0.964
E D  4FZAB                143    0.387  55.810   1.432   0.021   0.279   0.699
B V  4FZAB                144    0.033   5.087   0.437   0.018   0.846   0.136
B K  4FZAB                145    0.138  28.387   1.471   0.011   0.918   0.071
B L  4FZAB                146    0.034   6.244   0.417   0.011   0.918   0.071
B A  4FZAB                147    0.036   4.011  -0.180   0.018   0.846   0.136
B A  4FZAB                148    0.081   8.893  -0.962   0.079   0.592   0.329
B F  4FZAB                149    0.075  15.153  -0.046   0.216   0.235   0.548
B G  4FZAB                150    0.091   7.162  -1.115   0.453   0.248   0.299
B V  4FZAB                151    0.092  14.171   0.472   0.578   0.229   0.194
B A  4FZAB                152    0.032   3.559  -0.682   0.649   0.163   0.188
B G  4FZAB                153    0.222  17.495   0.614   0.725   0.163   0.112
B Q  4FZAB                154    0.295  52.758   0.719   0.662   0.225   0.113
B L  4FZAB                155    0.084  15.399   0.566   0.578   0.229   0.194
E T  4FZAB                156    0.370  51.374   0.126   0.502   0.102   0.396
E D  4FZAB                157    0.603  86.863  -0.165   0.268   0.043   0.689
E T  4FZAB                158    0.561  77.880  -0.683   0.268   0.043   0.689
E Q  4FZAB                159    0.529  94.497  -1.467   0.257   0.016   0.727
E I  4FZAB                160    0.474  87.764  -0.588   0.115   0.016   0.868
E K  4FZAB                161    0.419  86.106  -0.126   0.053   0.043   0.903
E R  4FZAB                162    0.303  69.479   0.032   0.052   0.084   0.864
E N  4FZAB                163    0.380  55.632   0.355   0.019   0.141   0.840
B T  4FZAB                164    0.072   9.931  -0.656   0.022   0.359   0.619
B F  4FZAB                165    0.078  15.675  -0.551   0.021   0.756   0.223
B V  4FZAB                166    0.074  11.420  -0.435   0.021   0.756   0.223
B G  4FZAB                167    0.040   3.124  -1.495   0.022   0.552   0.426
B T  4FZAB                168    0.042   5.770   0.499   0.004   0.197   0.799
B P  4FZAB                169    0.057   8.046  -0.417   0.019   0.141   0.840
B F  4FZAB                170    0.077  15.534  -0.298   0.056   0.142   0.802
B W  4FZAB                171    0.059  14.310  -0.266   0.066   0.296   0.638
B M  4FZAB                172    0.034   6.903  -0.255   0.135   0.317   0.548
B A  4FZAB                173    0.034   3.791   0.312   0.278   0.093   0.628
B P  4FZAB                174    0.053   7.478   0.043   0.561   0.047   0.393
B E  4FZAB                175    0.087  15.164  -0.360   0.538   0.173   0.289
B V  4FZAB                176    0.126  19.428   0.245   0.594   0.285   0.121
B I  4FZAB                177    0.075  13.856   0.487   0.478   0.309   0.214
E Q  4FZAB                178    0.516  92.211   1.064   0.307   0.165   0.527
E Q  4FZAB                179    0.731 130.646  -0.408   0.113   0.043   0.844
E S  4FZAB                180    0.555  65.093   0.379   0.018   0.047   0.935
E A  4FZAB                181    0.595  65.591  -1.186   0.018   0.047   0.935
B Y  4FZAB                182    0.191  40.859  -0.230   0.018   0.047   0.935
E D  4FZAB                183    0.558  80.393  -0.325   0.018   0.019   0.964
E S  4FZAB                184    0.340  39.860   0.250   0.053   0.043   0.903
E K  4FZAB                185    0.340  69.938   0.837   0.191   0.086   0.723
B A  4FZAB                186    0.028   3.119   0.832   0.268   0.043   0.689
B D  4FZAB                187    0.054   7.839   0.313   0.455   0.046   0.498
B I  4FZAB                188    0.030   5.569   0.722   0.751   0.050   0.199
B W  4FZAB                189    0.046  10.991   0.142   0.831   0.044   0.125
B S  4FZAB                190    0.034   3.997  -0.902   0.938   0.007   0.055
B L  4FZAB                191    0.043   7.782   0.190   0.938   0.007   0.055
B G  4FZAB                192    0.037   2.873  -0.412   0.938   0.007   0.055
B I  4FZAB                193    0.026   4.902   0.769   0.938   0.007   0.055
B T  4FZAB                194    0.039   5.465   0.291   0.975   0.003   0.022
B A  4FZAB                195    0.019   2.127   1.286   0.970   0.001   0.030
B I  4FZAB                196    0.043   7.955   0.987   0.988   0.000   0.012
B E  4FZAB                197    0.033   5.852  -0.024   0.970   0.001   0.030
B L  4FZAB                198    0.034   6.170   0.405   0.970   0.001   0.030
B A  4FZAB                199    0.038   4.177   0.157   0.858   0.002   0.139
B K  4FZAB                200    0.167  34.434  -0.538   0.502   0.002   0.495
E G  4FZAB                201    0.268  21.107  -0.873   0.053   0.005   0.942
E E  4FZAB                202    0.338  59.049   0.446   0.016   0.005   0.979
B P  4FZAB                203    0.236  33.545   0.468   0.016   0.005   0.979
B P  4FZAB                204    0.097  13.693  -1.595   0.003   0.003   0.994
B N  4FZAB                205    0.116  17.012  -0.287   0.003   0.003   0.994
E S  4FZAB                206    0.438  51.345  -0.267   0.003   0.003   0.994
E D  4FZAB                207    0.761 109.631  -0.682   0.003   0.003   0.994
E M  4FZAB                208    0.316  63.172  -0.125   0.016   0.005   0.979
E H  4FZAB                209    0.464  84.420  -0.079   0.016   0.005   0.979
B P  4FZAB                210    0.145  20.561  -0.510   0.923   0.002   0.076
B M  4FZAB                211    0.261  52.146   0.128   0.970   0.001   0.030
E R  4FZAB                212    0.357  81.845   1.338   0.970   0.001   0.030
B V  4FZAB                213    0.029   4.473   0.169   0.970   0.001   0.030
B L  4FZAB                214    0.048   8.825   0.299   0.970   0.001   0.030
B F  4FZAB                215    0.253  50.717   0.789   0.970   0.001   0.030
B L  4FZAB                216    0.243  44.420   1.485   0.970   0.001   0.030
B I  4FZAB                217    0.031   5.809   0.271   0.923   0.002   0.076
B P  4FZAB                218    0.259  36.724  -0.060   0.694   0.003   0.303
E K  4FZAB                219    0.705 145.121   0.405   0.246   0.004   0.750
E N  4FZAB                220    0.605  88.499  -0.670   0.053   0.005   0.942
E N  4FZAB                221    0.684 100.152  -0.287   0.003   0.003   0.994
E P  4FZAB                222    0.299  42.400  -0.206   0.003   0.003   0.994
E P  4FZAB                223    0.290  41.123  -0.301   0.003   0.003   0.994
E T  4FZAB                224    0.649  90.030   0.613   0.003   0.003   0.994
B L  4FZAB                225    0.094  17.193  -0.071   0.003   0.003   0.994
E V  4FZAB                226    0.331  50.813   0.577   0.053   0.005   0.942
E G  4FZAB                227    0.835  65.714  -0.270   0.321   0.003   0.675
E D  4FZAB                228    0.553  79.673   1.151   0.406   0.004   0.590
B F  4FZAB                229    0.063  12.604   0.963   0.406   0.004   0.590
B T  4FZAB                230    0.096  13.246   0.024   0.181   0.016   0.803
E K  4FZAB                231    0.684 140.802   1.045   0.923   0.002   0.076
E S  4FZAB                232    0.343  40.188   1.132   0.970   0.001   0.030
B F  4FZAB                233    0.017   3.452   1.154   0.970   0.001   0.030
E K  4FZAB                234    0.285  58.666   1.633   0.970   0.001   0.030
E E  4FZAB                235    0.465  81.236   1.611   0.970   0.001   0.030
B F  4FZAB                236    0.046   9.152   0.850   0.970   0.001   0.030
B I  4FZAB                237    0.023   4.200   1.153   0.988   0.000   0.012
E D  4FZAB                238    0.330  47.481   1.860   0.970   0.001   0.030
E A  4FZAB                239    0.340  37.479   1.998   0.970   0.001   0.030
B C  4FZAB                240    0.024   3.412   0.872   0.923   0.002   0.076
B L  4FZAB                241    0.043   7.782   0.245   0.858   0.002   0.139
E N  4FZAB                242    0.468  68.515   1.537   0.694   0.003   0.303
E K  4FZAB                243    0.382  78.557   1.441   0.321   0.003   0.675
B D  4FZAB                244    0.211  30.362   0.563   0.016   0.005   0.979
B P  4FZAB                245    0.125  17.723  -0.129   0.016   0.005   0.979
E S  4FZAB                246    0.703  82.415   0.397   0.016   0.005   0.979
E F  4FZAB                247    0.453  90.997   0.136   0.018   0.019   0.964
B R  4FZAB                248    0.209  47.930  -0.570   0.018   0.019   0.964
B P  4FZAB                249    0.224  31.814  -0.198   0.115   0.016   0.868
E T  4FZAB                250    0.278  38.614   1.048   0.058   0.017   0.925
B A  4FZAB                251    0.038   4.133  -0.582   0.923   0.002   0.076
E K  4FZAB                252    0.463  95.260   1.576   0.923   0.002   0.076
E E  4FZAB                253    0.330  57.634   1.196   0.858   0.002   0.139
B L  4FZAB                254    0.040   7.416   0.294   0.858   0.002   0.139
B L  4FZAB                255    0.100  18.365   0.329   0.694   0.003   0.303
E K  4FZAB                256    0.673 138.539   0.761   0.406   0.004   0.590
B H  4FZAB                257    0.165  29.959   1.437   0.176   0.004   0.820
E K  4FZAB                258    0.476  98.016   0.471   0.430   0.016   0.555
B F  4FZAB                259    0.133  26.713   0.177   0.660   0.049   0.291
B I  4FZAB                260    0.049   9.046   0.189   0.701   0.107   0.192
E V  4FZAB                261    0.342  52.489   1.320   0.660   0.049   0.291
E K  4FZAB                262    0.578 118.853   0.752   0.561   0.047   0.393
E N  4FZAB                263    0.334  48.956   0.405   0.339   0.016   0.645
E S  4FZAB                264    0.333  38.969  -0.387   0.181   0.016   0.803
E K  4FZAB                265    0.639 131.339   0.001   0.058   0.017   0.925
E K  4FZAB                266    0.526 108.157  -0.311   0.115   0.016   0.868
E T  4FZAB                267    0.506  70.141  -0.609   0.257   0.016   0.727
E S  4FZAB                268    0.548  64.202  -0.785   0.406   0.004   0.590
E Y  4FZAB                269    0.377  80.586   0.351   0.321   0.003   0.675
B L  4FZAB                270    0.058  10.601   0.108   0.782   0.003   0.216
E T  4FZAB                271    0.406  56.257   1.706   0.923   0.002   0.076
E E  4FZAB                272    0.571  99.824   1.704   0.970   0.001   0.030
B L  4FZAB                273    0.107  19.628   1.067   0.970   0.001   0.030
B I  4FZAB                274    0.050   9.158   0.523   0.970   0.001   0.030
E D  4FZAB                275    0.549  79.082   1.814   0.970   0.001   0.030
E R  4FZAB                276    0.442 101.287   1.707   0.970   0.001   0.030
B F  4FZAB                277    0.069  13.828   0.772   0.970   0.001   0.030
E K  4FZAB                278    0.421  86.538   0.960   0.923   0.002   0.076
E R  4FZAB                279    0.664 151.987   1.627   0.858   0.002   0.139
B W  4FZAB                280    0.230  55.315   0.536   0.694   0.003   0.303
E K  4FZAB                281    0.574 118.154   0.955   0.406   0.004   0.590
E A  4FZAB                282    0.797  87.796   0.498   0.109   0.005   0.886
E E  4FZAB                283    0.878 153.369   1.232   0.003   0.003   0.994

Explain the output.  Go back.
```

---
